# The role of preoperative inflammatory markers in patients with central nervous system tumors, focus on glioma

**DOI:** 10.3389/fonc.2022.1055783

**Published:** 2022-11-22

**Authors:** Fan Chen, Min Chao, Tao Huang, Shaochun Guo, Yulong Zhai, Yuan Wang, Na Wang, Xuan Xie, Liang Wang, Peigang Ji

**Affiliations:** ^1^ Department of Neurosurgery, Tangdu Hospital of Fourth Military Medical University, Xi’an, China; ^2^ Reproductive Medicine Center, Department of Gynecology & Obstetrics, Xijing Hospital of Fourth Military Medical University, Xi’an, China

**Keywords:** inflammatory markers, glioma, meningioma, IDH status, diagnosis, molecular typing

## Abstract

**Background:**

CNS tumors, particularly gliomas, are associated with a high rate of disability and lethality, and are typically diagnosed with histopathology and immunohistochemistry. Our research aims to develop a minimally invasive method for diagnosing, grading and molecular typing glioma.

**Methods:**

We collected patients who underwent surgery for glioma, Trigeminal neuralgia/Hemifacial spasm, schwannoma, pituitary adenomas and meningioma at our hospital from June 2019 to June 2021. Preoperative WBCs, neutrophils, lymphocytes, monocytes, platelet counts and albumin levels were collected. Preoperative NLR, dNLR, PLR, LMR and PNI were calculated, and the correlation between them and glioma diagnosis as well as grading was analyzed. We also evaluated the diagnostic significance of NLR, dNLR, PLR, LMR, PNI and their combinations for gliomas, particularly GBM, as well as the diagnostic significance of IDH molecular typing of gliomas.

**Results:**

There were 182 healthy samples and 3101 diseased samples in our study. Compared with other groups, glioma patients had significantly higher preoperative NLR, dNLR and PLR values, but lower LMR and PNI values. Further analysis showed that NLR, dNLR, and PLR were positively correlated with glioma grading, while LMR and PNI were negatively correlated with glioma grading. For the diagnosis of glioma, NLR showed a maximum AUC value of 0.8099 (0.7823-0.8374). For GBM, NLR showed a maximum AUC value of 0.9585 (0.9467-0.9703). In the combination, NLR+dNLR showed the highest AUC value of 0.8070(0.7849-0.8291). NLR showed significant statistical significance in all grades of glioma IDH molecular typing, while PLR did not show statistical significance.

**Conclusions:**

NLR has the greatest value for the diagnosis, differential diagnosis, grading and molecular typing of gliomas. The NLR+dNLR combination also showed high sensitivity and specificity. We believe that inflammatory parameters may serve as economical and specific markers for glioma diagnosis, grading, molecular typing, and progression.

## Introduction

Brain tumors and other central nervous system (CNS) tumors are considered one of the deadliest human cancers, with considerable morbidity and mortality in the United States (1). The latest version of the Central Brain Tumor Registry of the United States (CBTRUS) data showed that from 2014 to 2018, the number of deaths due to malignant brain tumors and other CNS tumors in the United States was 83,029, with average annual death of 16,606 and an average annual mortality rate of 4.43/100,000 (2). Among them, glioblastoma (GBM) is the most common malignant brain tumor, accounting for 14.3% of all brain tumors and 49.1% of malignant brain tumors, and meningioma is the most frequent non-malignant brain tumor, accounting for 39% of all brain tumors and 54.5% of non-malignant brain tumors (2). Glioma is the most common primary brain tumor (3, 4), accounting for 81% of CNS tumors (5), and GBM is the most aggressive and most common type of glioma (2). Despite the use of a combination of surgery, radiotherapy, immunotherapy, and Tumor Treating Fields, the 5-year survival rate for GBM is still poor (6–9). Unlike peripheral cancers, gliomas do not have specific or sensitive serum markers for detecting tumorigenesis, tumor grading, monitoring treatment response, and assessing prognosis (10–13). Histopathological examination has long been considered the gold standard for glioma diagnosis, while imaging is a complementary procedure for preoperative evaluation of glioma and postoperative monitoring of treatment response and tumor recurrence (14). Current examinations, on the other hand, are not only less sensitive and more expensive, but may also cause neurological damage or intracranial hemorrhage. Therefore, there is an urgent need to develop sensitive, cost-effective, and specific markers for glioma diagnosis, grading, and monitoring of treatment response and recurrence.

Multiple studies have confirmed the role of inflammatory responses in solid tumor pathogenesis, which may be closely related to tumor oncogenesis, progression, treatment resistance and prognosis (15–19). Inflammation in the host leads to changes in circulating levels of the inflammatory markers white blood cells (WBCs), neutrophils, lymphocytes, monocytes, platelet counts and albumin. These serum indicators are often economical and readily available as part of normal preoperative testing. Currently, studies have confirmed the predictive role of circulating inflammatory markers and their ratios in solid tumors, including lung cancer (20), penile cancer (21), cervical cancer (22), colorectal cancer (23), thyroid carcinoma (24), bladder cancer (25), esophageal squamous cell carcinoma (26), Hilar Cholangiocarcinoma (27), breast cancer (28), et al. Compared to peripheral solid tumors, only a limited number of studies have reported the diagnostic value of circulating inflammatory markers in gliomas (3, 29, 30). Moreover, most relevant studies have focused on survival and recurrence in GBM patients, and there are no in-depth studies on inflammatory markers and their ratios in the diagnosis, grading and molecular typing of glioma, and the reported studies have not reached uniform conclusions.

We analyzed the differences in levels of representative inflammatory markers between glioma patients and patients with trigeminal neuralgia/hemifacial spasm, schwannoma, pituitary adenomas, meningiomas, as well as healthy controls; and further evaluated the validity of NLR (ratio of neutrophil/lymphocyte), dNLR (derived NLR), PLR (ratio of platelet/lymphocyte), LMR (ratio of lymphocyte/monocyte), and PNI (prognostic nutritional index) and their combinations in the diagnosis, grading, and molecular typing of gliomas.

## Materials and methods

We collected medical records of patients with newly diagnosed glioma, schwannoma, meningioma, Pituitary Adenomas, or trigeminal neuralgia/hemifacial spasm at Tangdu Hospital from Jan 2019 to Dec 2021, and analyzed them retrospectively. Our study was approved by the Ethics Committee of Tangdu Hospital.

### Healthy controls and patients

The following criteria must be met by all patients included in this study: (1) postoperative histologically verified Schwannoma, Pituitary Adenomas and Meningiomas, the diagnostic criteria for trigeminal neuralgia (TN) were based on the ICHD-3β criteria and the 2018 update, Hemifacial spasm (HS) diagnosed by the functional neurosurgeon, Gliomas that were histologically verified and graded according to WHO diagnostic criteria; (2) Without other peripheral tumors, only one CNS tumor present; (3) Without pre-operative adjuvant treatment such as radiotherapy, chemotherapy or immunotherapy and other cancer-specific treatments; (4) With complete data on preoperative blood counts and serum albumin levels; (5) No preoperative conditions that could alter the levels of blood parameters, such as hematological disorders, inflammatory diseases, metabolic syndrome, autoimmune diseases, fever, hyperlipidemia, et al, and no antibiotics were used; (6) informed consent. As the healthy control group, we reviewed the medical records of healthy individuals who underwent their annual health checks at our hospital.

### Data collection

Information regarding demographic and clinicopathological variables of the included samples, including age, gender, tumor type, tumor grade, molecular subtype and histological type was retrieved from the patient’s medical records. Preoperative blood samples were collected from patients as part of the standard preoperative examination. Blood samples were routinely collected within 3 days prior to surgery. All blood tests were performed by the laboratory department at our hospital. WBC, lymphocyte, neutrophil, monocyte and platelet counts were collected by routine blood tests, and patient albumin levels were collected by hepatic function tests.

### Data processing

We calculated the ratio of neutrophil count to lymphocyte count as NLR, the quotient of (WBC count minus neutrophil count) to lymphocyte count as dNLR, the ratio of platelet count to lymphocyte count as PLR, the ratio of lymphocyte count to monocyte count as LMR, and albumin count [g/L] + total lymphocyte count × 5 as nutritional index PNI.

### Statistical analysis

Statistical analysis of this study was performed using SPSS version 22.0. And all of our data were presented as median and range. The Kruskal-Wallis test was used to determine the correlation of preoperative inflammatory markers NLR, dNLR, PLR, LMR, and PNI with tumor type and tumor grade. Group comparisons of nonparametric data were performed using the Mann-Whitney U test. The correlation between the two variables was performed by Pearson correlation test. The diagnostic performance of preoperative inflammatory markers and their combinations in receivers was assessed by calculating the area under the curve (AUC) obtained from the receiver operating characteristic (ROC) curve. p < 0.05 was considered statistically significant.

## Results

### General information

Finally, 182 healthy individuals, 96 TN/HS patients, 316 patients with schwannoma, 357 patients with pituitary adenomas, 1271 patients with meningiomas, and 1061 patients with gliomas were included in the analysis. **Table 1** shows the detailed demographic information of the samples included in this study. The median age of the healthy control samples was 41.9 (6-85) years and 101 of them (55.49%) were male, 81 of them (44.51%) were female. The median age of the patients with TN or HS was 52.9(21-83) years and 44 of them (45.83%) were male, 52 of them (54.17%) were female. For the patients with schwannoma, the median age was 48.6(14-85) years and 144 of them (45.57%) were male, 172 of them (54.43%) were female. As for pituitary adenomas patients, the median age was 43.6(16-77) years and 185 of them (51.82%) were male, 172 of them (48.18%) were female. The median age of meningioma samples was 51.5(16-85), and 327 of them (25.73%) were male, 944 of them (74.27%) were female. For the samples of glioma, the median age was 46.4(3-87) years and 620 of them (58.44%) were male, 441 of them (41.56%) were female. 103 glioma patients were further classified as WHO I, 270 patients as WHO II, 241 patients as WHO III, and 447 patients as WHO IV.

**Table 1:** Preoperative characteristics of patients with central nervous system tumors and healthy controls.

	Healthy Controls	TN/HS	Schwannoma	Pituitary Adenomas	meningioma	Glioma
Age	41.9 (6-85)	52.9 (21-83)	48.6 (14-85)	43.6 (16-77)	51.5 (16-85)	46.4 (3-87)
Patients (n)	182	96	316	357	1271	1061
Male (n)	101	44	144	185	327	620
Female (n)	81	52	172	172	944	441
WBCs (x10^9^/L)	6.21 (3.51-9.43)	6.28 (3.67-13.96)	5.81 (2.93-9.62)	6.11 (2.98-10.58)	5.89 (2.75-15.44)	7.15 (2.85-22.44)^α-ϵ^
Neutrophils (x10^9^/L)	3.25 (1.79-6.12)	3.29 (2.01-6.31)	3.17 (1.79-6.31)	3.25 (1.79-6.63)	3.41 (1.32-12.21)^γ^	4.24 (0.83-18.21)^α-ϵ^
Lymphocytes (x10^9^/L)	2.26 (1.21-3.82)	2.29 (1.44-3.79)	2.18 (2.21-3.82)	2.20 (1.23-3.90)	1.96 (0.81-4.30)^α-δ^	1.96 (0.63-3.67)^α-δ^
Monocytes (x10^9^/L)	0.37 (0.13-0.78)	0.36 (0.17-0.75)	0.36 (0.12-0.78)	0.37 (0.13-0.80)	0.37 (0.11-0.98)	0.41 (0.11-0.98)^α-ϵ^
Plateles (x10^9^/L)	242.1 (132-360)	242.7 (138-351)	241.3 (132-360)	253.1 (138-378)	240 (101-503)^δ^	239 (75.31-503)^δ^
Albumin (g/L)	42.8 (35.2-54.5)	42.5 (34.1-54.5)	43.15 (34.1-59.5)	41.86 (32.39-56.95)	42.8 (31.9-54.9)^δ^	38.7 (23.5-53.1)^α-ϵ^
NLR	1.49 (0.71-2.88)	1.49 (0.72-2.32)	1.51 (0.64-2.72)	1.53 (0.63-2.79)	1.83 (0.77-7.47)^α-δ^	2.29 (0.58-11.14)^α-ϵ^
dNLR	1.34 (0.52-2.57)	1.35 (0.52-4.77)	1.25 (0.28-3.06)	1.33 (0.27-3.44)	1.32 (0.36-6.64)	1.54 (0.15-6.64)^α,γ-ϵ^
PLR	112.10 (54.33-228.08)	111.43 (54.32-225.18)	116.25 (54.33-255.45)	120.25 (53.43-262.97)	130.76 (46.83-343.64)^α-δ^	131.22 (35.87-363.64)^α-δ^
LMR	6.83 (1.95-13.86)	6.99 (1.85-13.79)	6.62 (1.75-13.63)	6.62 (1.73-14.14)	5.88 (1.30-13.23)^α-δ^	5.28 (1.04-11.90)^α-ϵ^
PNI	54.12 (41.25-73.60)	53.95 (43.30-67.41)	54.05 (41.25-73.60)	53.11 (39.61-72.76)	52.60 (39.18-73.32)^α,γ^	48.55 (26.66-73.32)^α-ϵ^

TN/HS, Trigeminal neuralgia/Hemifacial spasm.

^α^ p < 0.05 vs healthy controls.

^β^ p < 0.05 vs TN/HS.

^γ^ p < 0.05 vs Schwannoma.

^δ^ p < 0.05 vs Pituitary Adenomas.

^ϵ^ p < 0.05 vs meningioma.

### Comparison of various preoperative inflammatory markers in healthy control and tumor groups

Patients with TN/HS, schwannoma, and pituitary adenomas showed no significant differences from healthy control groups in laboratory tests for WBCs, neutrophils, lymphocytes, monocytes, platelets, and albumin. We observed that in glioma patients, preoperative WBCs, neutrophil and monocyte counts were significantly higher than in other groups, while lymphocyte counts and albumin levels were significantly lower. Interestingly, we found a decrease in preoperative WBC count in patients with meningioma, as well as similar changes in neutrophil count and lymphocyte count as in glioma (**Table 1**). Surprisingly, no significant differences in platelet counts were observed between all groups.

**Figure 1:**
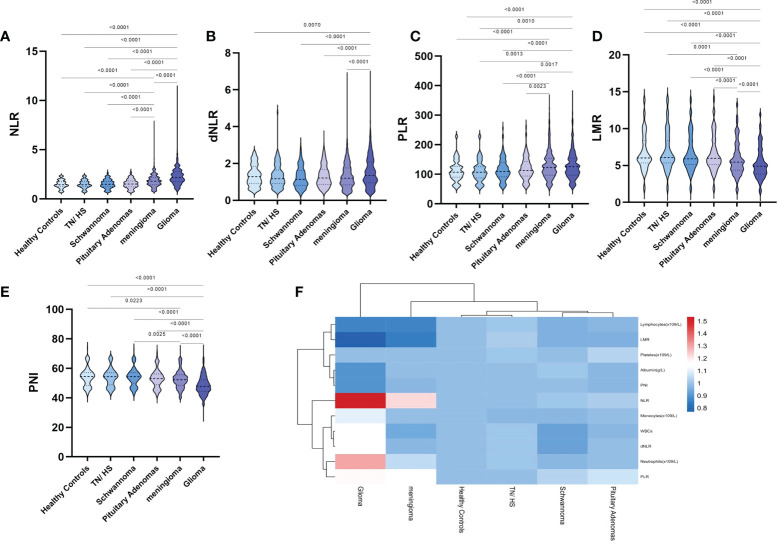
Violin diagram showing comparative results of preoperative inflammatory markers in the healthy control group, the trigeminal neuralgia/hemifacial spasm group, schwannoma group, pituitary adenomas group, meningioma group, and glioma group. **(A)** NLR, **(B)** dNLR, **(C)** PLR, **(D)** LMR, **(E)** PNI, **(F)** Heat map of inflammatory marker features between the groups. The dashed line in the middle represents the median and the dashed lines on both sides represent the interquartile range.

We also further processed the preoperative laboratory parameters, and we found no significant differences in NLR, dNLR, PLR, LMR and PNI in the TH/NS, schwannoma and pituitary adenoma groups compared to healthy controls (**Figures 1A–F**). Compared to other groups, NLR (**Figure 1A**), dNLR (**Figure 1B**) and PLR (**Figure 1C**) were significantly increased in patients with glioma, but LMR (**Figure 1D**) and nutritional index PNI (**Figure 1E**) were significantly decreased. Unexpectedly, meningiomas showed a similar trend in NLR, PLR, LMR and PNI to gliomas, but the degree of change was not as significant as gliomas.

### Comparison of preoperative inflammatory markers in different glioma grades

Given that preoperative laboratory parameters and their further processing showed significant differences between gliomas and other groups, we further analyzed the relationship between relevant parameters and WHO classification of glioma (**Table 2**). Among laboratory parameters, patients with glioma WHO IV had significantly higher WBCs, neutrophils, and monocyte counts (p value < 0.05), and significantly lower lymphocyte counts and albumin levels compared to healthy controls (p value < 0.05). Meanwhile, WBCs and neutrophil counts were significantly higher in patients with grade IV compared with other grades of glioma, while lymphocyte and monocyte counts and albumin levels were not significantly different. Unexpectedly, we found significantly lower platelet counts in patients with WHO grade I glioma compared to healthy controls, but no significant difference in platelet counts in patients with WHO grade IV glioma compared to healthy controls.

**Table 2:** Correlations between preoperative inflammatory markers and WHO grade in glioma patients.

	Healthy Controls	Glioma			
		WHO I	WHO II	WHO III	WHO IV
WBCs (x10^9^/L)	6.21 (3.51-9.43)	6.35 (2.86-10.53)	6.71 (3.25-10.83)	6.44 (3.42-11.78)	7.88 (3.32-22.44)^α-γ^
Neutrophils (x10^9^/L)	3.25 (1.79-6.12)	3.66 (0.83-5.29)	4.06 (2.10-8.90)^α^	3.90 (2.12-7.49)	4.67 (1.30-18.21)^α-γ^
Lymphocytes (x10^9^/L)	2.26 (1.21-3.82)	1.90 (0.63-2.84)	1.92 (0.82-3.16)	1.94 (0.87-3.67)	2.01 (0.81-3.67)
Monocytes (x10^9^/L)	0.37 (0.13-0.78)	0.41 (0.13-0.76)	0.40 (0.14-0.84)	0.44 (0.20-0.98)	0.40 (0.11-0.98)
Plateles (x10^9^/L)	242 (132-360)	220 (75-337)	232 (130-356)	242 (131-356)^α^	247 (101-503)^α-β^
Albumin (g/L)	42.8 (35.2-54.5)	38.0 (23.5-47.8)	38.3 (29.3-53.9)	39.4 (27.8-54.9)	38.8 (29.3-54.9)
NLR	1.49 (0.71-2.88)	1.67 (0.78-4.77)	1.87 (0.80-4.88)	2.06 (0.79-5.03)^α-β^	2.81 (0.59-11.14)^α-γ^
dNLR	1.34 (0.52-2.57)	1.24 (0.39-2.83)	1.31 (0.37-4.57)	1.39 (0.36-3.81)	1.83 (0.16-6.64)^α-γ^
PLR	112.10 (54.33-228.08)	114.76 (52.45-265.65)	124.84 (54.29-363.64)	128.47 (55.86-327.26)	140.28 (35.87-359.99)^α-γ^
LMR	6.83 (1.95-13.86)	6.31 (2.78-11.90)	5.80 (1.40-11.90)	5.16 (1.04-11.87)^α-β^	4.77 (1.16-10.39)^α-β^
PNI	54.12 (41.25-73.60)	53.44 (34.88-67.31)	49.81 (34.89-66.70)^α^	48.37 (35.65-65.71)^α-β^	46.75 (26.67-73.32)^α-γ^

^α^ p < 0.05 vs WHO I.

^β^ p < 0.05 vs WHO II.

^γ^ p < 0.05 vs WHO III.

**Figure 2:**
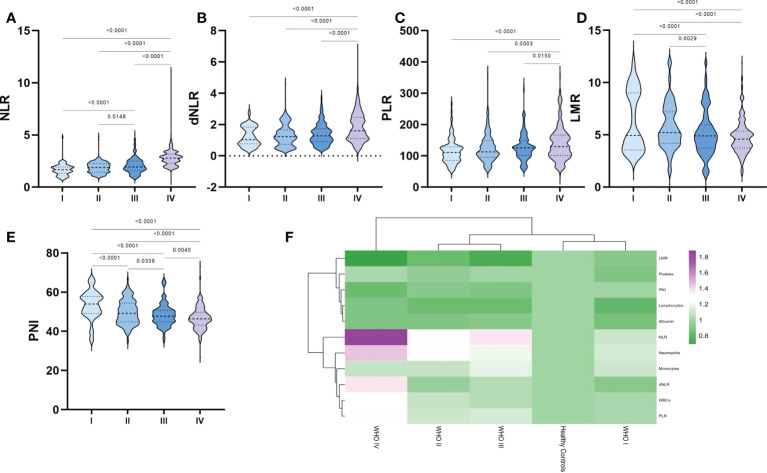
Violin diagram showing comparative results of preoperative inflammatory markers in different grades of glioma groups. **(A)** NLR, **(B)** dNLR, **(C)** PLR, **(D)** LMR, **(E)** PNI, **(F)** Heat map of inflammatory marker profiles in different grades of glioma and healthy controls. The dashed line in the middle represents the median and the dashed lines on both sides represent the interquartile range.

After further processing of laboratory parameters, we analyzed the differences in the levels of NLR, dNLR, PLR, LMR and PNI between the different glioma grades (**Figures 2A–F**). Our results showed statistically significant differences between WHO grade IV gliomas and other grades of gliomas in terms of NLR, dNLR, PLR, LMR and PNI, with significantly higher NLR (**Figure 2A**), dNLR (**Figure 2B**) and PLR (**Figure 2C**), and significantly lower LMR (**Figure 2D**) and PNI (**Figure 2E**). NLR showed a trend of closely correlated changes with glioma grade, with grade III being significantly higher than grade I-II and grade IV being significantly higher than grade III. LMR and PNI showed a decreasing trend closely related to glioma grade, with grade III significantly lower than grade I-II and grade IV significantly lower than grade III. Surprisingly, although no positive correlation was observed between platelet count and glioma grade, there was a positive correlation between PLR and glioma grade (**Figure 2C**).

### Correlation of preoperative inflammatory markers with healthy control and glioma grade

**Figure 3:**
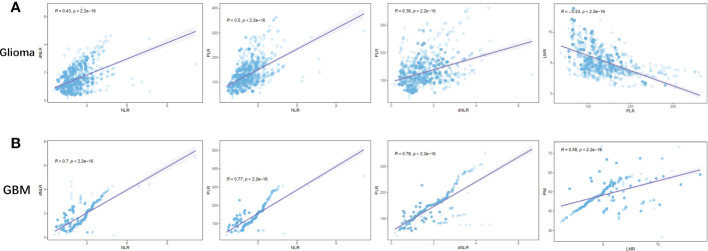
Correlation between preoperative inflammatory markers in patients with glioma. **(A)** NLR vs dNLR, NLR vs PLR, dNLR vs PLR, PLR vs LMR in Glioma; **(B)** NLR vs dNLR, NLR vs PLR, dNLR vs PLR, LMR vs PNI in GBM.

To further investigate the relationship between inflammatory markers and glioma grade, we analyzed the correlation of NLR, dNLR, PLR, LMR, and PNI with each other in glioma and GBM (**Figure 3**, **Figure S1**). In glioma, we found that positive correlations were shown between NLR and dNLR (R=0.43, p<0.0001), NLR and PLR (R=0.50, p<0.0001), and dNLR and PLR (R=0.36, p<0.0001), respectively (**Figure 3A**), while negative correlations were shown between PLR and LMR (**Figure 3A**, R=-0.53, p<0.0001), and NLR and PNI (**Figure S1A**, R=-0.42, p<0.0001), respectively. In GBM, we found that positive correlations were shown between NLR and dNLR (R=0.70, p<0.0001), NLR and PLR (R=0.77, p<0.0001), dNLR and PLR (R=0.78, p<0.0001), and LMR and PNI (R=0.48, p<0.0001), respectively (**Figure 3B**). Although NLR and dNLR, NLR and PLR, and dNLR and PLR showed positive correlations in both the glioma and GBM groups, the correlations were significantly higher in the GBM group than in the glioma group.

### Diagnostic value of preoperative inflammatory markers in glioma diagnosis and glioma grading

**Figure 4:**
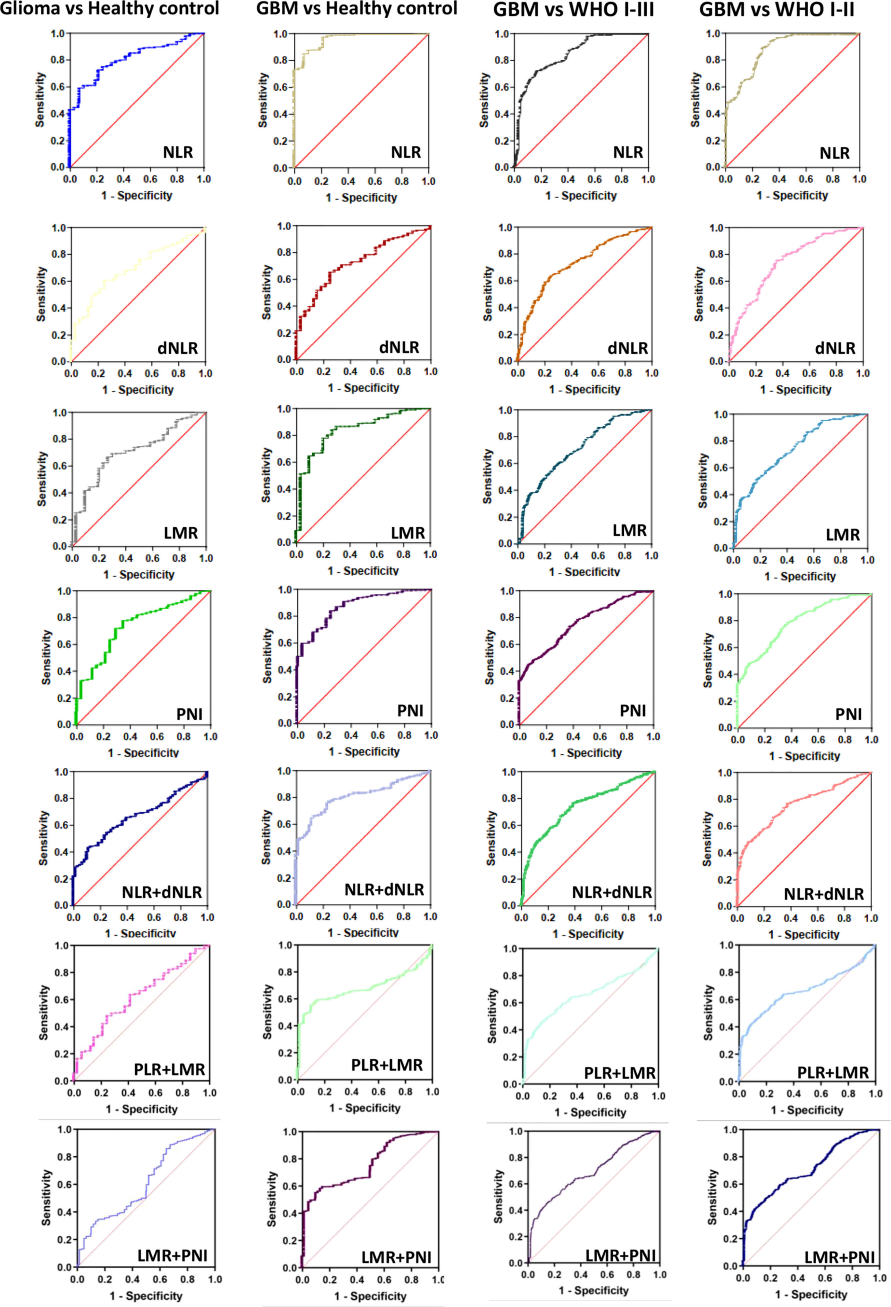
The diagnostic value of preoperative inflammatory markers in glioma diagnosis and glioma grading. Glioma vs Healthy control, GBM vs Healthy control, GBM vs WHO I-III, GBM vs WHO I-II.

We next further explored the efficacy of NLR, dNLR, PLR, LMR and PNI for the diagnosis and grading of gliomas (**Figure 4**, **Figure S2**, **Table 3**). When comparing glioma patients with healthy controls, the AUC for NLR was 0.8099 (0.7823-0.8374), for dNLR was 0.6914 (0.6491-0.7337), for PLR was 0.5213 (0.4948-0.5477), for LMR was 0.7045 (0.6663-0.7428), and AUC for PNI was 0.7385 (0.7016-0.7754). The AUC was 0.9585(0.9467-0.9703) for NLR, 0.7291(0.6918-0.7665) for dNLR, 0.5538(0.5158-0.5919) for PLR, 0.8376(0.8054-0.8698) for LMR, and 0.8736(0.8481-0.8991) for PNI when patients with GBM were tested against healthy controls. The AUC was 0.8424(0.8189-0.8660) for NLR, 0.7300(0.7026-0.7574) for dNLR, 0.6084(0.5768-0.6399) for PLR, 0.7071(0.6790-0.7352) for LMR, and 0.7574(0.7317-0.7832) for PNI when patients with GBM were tested against WHO I-III. The AUC was 0.8789(0.8562-0.9015) for NLR, 0.739(0.7087-0.7700) for dNLR, 0.5959(0.5622-0.6296) for PLR, 0.7386(0.7077-0.7695) for LMR, and 0.7844(0.7570-0.8119) for PNI when patients with GBM were tested against WHO I-II. NLR had the highest accuracy in predicting glioma in multiple analyses, with the highest AUC of 0.9585 when comparing glioma patients with healthy controls.

**Table 3:** Diagnostic value of various inflammatory markers and their combinations in gliomas and their grades.

	AUC (95%)			
Maker	Glioma vs Healthy Controls	GBM vs Healthy Controls	GBM vs Glioma I-III	GBM vs Glioma I-II
NLR	0.8099 (0.7823-0.8374)	0.9585 (0.9467-0.9703)	0.8424 (0.8189-0.8660)	0.8789 (0.8562-0.9015)
dNLR	0.6914 (0.6491-0.7337)	0.7291 (0.6918-0.7665)	0.7300 (0.7026-0.7574)	0.739 (0.7087-0.7700)
PLR	0.5213 (0.4948-0.5477)	0.5538 (0.5158-0.5919)	0.6084 (0.5768-0.6399)	0.5959 (0.5622-0.6296)
LMR	0.7045 (0.6663-0.7428)	0.8376 (0.8054-0.8698)	0.7071 (0.6790-0.7352)	0.7386 (0.7077-0.7695)
PNI	0.7385 (0.7016-0.7754)	0.8736 (0.8481-0.8991)	0.7574 (0.7317-0.7832)	0.7844 (0.7570-0.8119)
NLR+dNLR	0.6659 (0.6414-0.6904)	0.8070 (0.7849-0.8291)	0.7348 (0.7144-0.7552)	0.7560 (0.7345-0.7776)
NLR+PLR	0.6073 (0.5732-0.6414)	0.616 (0.5773-0.6547)	0.5601 (0.5365-0.5837)	0.5753 (0.5470-0.6035)
NLR+LMR	0.5290 (0.4883-0.5697)	0.5364 (0.4905-0.5823)	0.5427 (0.5188-0.5667)	0.5420 (0.5121-0.5720)
NLR+PNI	0.5178 (0.4762-0.5595)	0.5205 (0.4738-0.5672)	0.5156 (0.4914-0.5398)	0.5190 (0.4884-0.5495)
dNLR+PLR	0.5429 (0.5127-0.5732)	0.5617 (0.5259-0.5975)	0.5449 (0.5213-0.5685)	0.5512 (0.5234-0.5791)
dNLR+LMR	0.5386 (0.5026-0.5745)	0.5217 (0.4796-0.5638)	0.5195 (0.4956-0.5434)	0.5104 (0.4810-0.5399)
dNLR+PNI	0.5465 (0.5099-0.5832)	0.5338 (0.4912-0.5764)	0.5004 (0.4764-0.5244)	0.5051 (0.4753-0.5348)
PLR+LMR	0.6194 (0.5778-0.6610)	0.6769 (0.6519-0.7019)	0.6549 (0.6334-0.6764)	0.6612 (0.6382-0.6842)
PLR+PNI	0.5313 (0.5053-0.5572)	0.6899 (0.6659-0.7139)	0.6737 (0.6525-0.6950)	0.6773 (0.6549-0.6998)
LMR+PNI	0.6108 (0.5793-0.6422)	0.7569 (0.7314-0.7823)	0.6912 (0.6719-0.7124)	0.7083 (0.6862-0.7305)

To further analyze the sensitivity of inflammatory markers in glioma diagnosis and glioma grading, we combined inflammatory markers. The AUC was 0.6659(0.6414-0.6904) for NLR+dNLR, 0.6073(0.5732-0.6414) for NLR+PLR, 0.6194(0.5778-0.6610) for PLR+LMR, 0.5313(0.5053-0.5572) for PLR+PNI, and 0.6108(0.5793-0.6422) for LMR+PNI when patients with glioma were tested against healthy controls. When comparing GBM patients with healthy controls, the AUC was 0.8070(0.7849-0.8291) for NLR+dNLR, 0.616(0.5773-0.6547) for NLR+PLR, 0.6769(0.6519-0.7019) for PLR+LMR, 0.6899(0.6659-0.7139) for PLR+PNI, and 0.7569(0.7314-0.7823) for LMR+PNI. The AUC was 0.7348(0.7144-0.7552) for NLR+dNLR, 0.5601(0.5365-0.5837) for NLR+PLR, 0.6549(0.6334-0.6764) for PLR+LMR, 0.6737(0.6525-0.6950) for PLR+PNI, and 0.6912(0.6719-0.7124) for LMR+PNI, when patients with GBM were tested against WHO I-III. The AUC was 0.7560(0.7345-0.7776) for NLR+dNLR, 0.5753(0.5470-0.6035) for NLR+PLR, 0.6612(0.6382-0.6842) for PLR+LMR, 0.6773(0.6549-0.6998) for PLR+PNI, and 0.7083(0.6862-0.7305) for LMR+PNI, when patients with GBM were tested against WHO I-II. The best diagnostic value was obtained with the combination of NLR+dNLR and LMR+PNI.

### Correlation of preoperative inflammatory markers and IDH status in glioma

**Figure 5:**
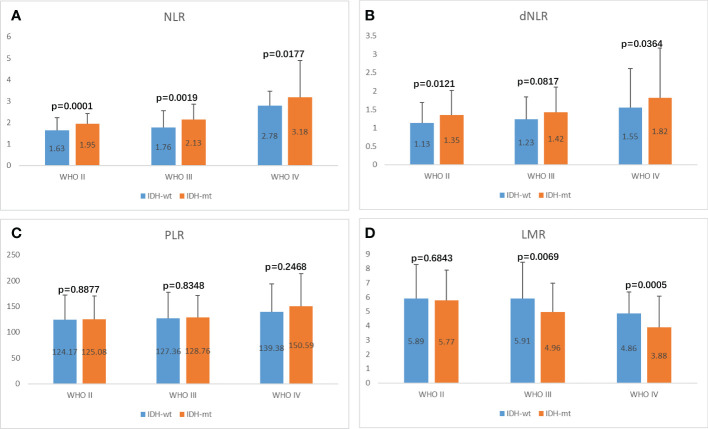
Potential influences on preoperative inflammatory markers caused by IDH1 mutation within glioma grade. **(A)** NLR, **(B)** dNLR, **(C)** PLR, **(D)** LMR.

Isocitrate dehydrogenase (IDH) status is an important basis for molecular typing of gliomas and is reflected in WHO 2016 revised diagnostic criteria (7, 31). We further analyzed the differences in inflammatory markers across IDH status in each glioma group to assess their diagnostic value in predicting IDH mutations and wild-type gliomas (**Figure 5**, **Figure S3**, **Table 4**). NLR demonstrated the best diagnostic value in WHO II IDH wild-type and IDH mutant gliomas (p-value < 0.0001; median 1.63 and 1.95, respectively), WHO III (p-value = 0.0019; median 1.76 and 2.13) and WHO IV gliomas (p-value 0.0177, median 2.78 and 3.18, respectively) were significantly different (**Figure 5A**). dNLR was significantly different in WHO grade II IDH wild-type and IDH mutant gliomas (p-value = 0.0121; median 1.13 and 1.35, respectively) and WHO IV gliomas (p-value 0.0177, median 2.78 and 3.18, respectively) (**Figure 5B**). LMR was significantly different in WHO III IDH wild-type and IDH mutant gliomas (p-value=0.0069; median 5.91 and 4.96, respectively) and grade IV gliomas (p-value 0.0005, median 4.86 and 3.88, respectively) (**Figure 5D**). PLR (**Figure 5C**) and PNI (**Figure S3**) did not show significant differences in different grades of IDH wild-type and IDH mutant gliomas.

**Table 4:** Influence of IDH status on inflammatory markers in different grades of glioma.

	WHO II			WHO III			WHO IV		
	wt	mt	p	wt	mt	p	wt	mt	p
NLR	1.63	1.95	0.0001	1.76	2.13	0.0019	2.78	3.18	0.0177
dNLR	1.13	1.35	0.0121	1.23	1.42	0.0817	1.55	1.82	0.0364
PLR	124.17	125.08	0.8877	127.36	128.76	0.8348	139.38	150.59	0.2468
LMR	5.89	5.77	0.6843	5.91	4.96	0.0069	4.86	3.88	0.0005
PNI	48.68	50.2	0.0775	46.78	48.75	0.0382	46.71	47.26	0.5674

wt, IDH-wildtype.

mt, IDH-mutant.

## Discussion

A growing number of studies have implicated chronic inflammation in cancer oncogenesis and development, and several inflammatory parameters, including: neutrophils, lymphocytes, platelets, monocytes, and albumin have been reported to aid in the diagnosis of a variety of cancers (3, 32–36). It was reported that in patients with metastatic non-small cell lung cancer, elevated pre-treatment NLR and PLR were associated with overall survival (OS), progression-free survival (PFS), and treatment response rate (37). Elevated NLR was associated with lower OS, PFS, and cancer-specific survival in investigations with upper urothelial carcinoma, urothelial bladder cancer, and metastatic and advanced disease (38). Low Pre-treatment LMR has also been reported as a poor prognostic factor for OS in patients with rectal cancer who were treated with preoperative radiotherapy and chemotherapy (39). Although studies have confirmed the relationship between inflammatory parameters and GBM, most studies have focused on the prognosis of GBM. For example, McNamara et al. (40). revealed that preoperative NLR >4 in GBM patients had a poor prognosis and short survival. Although there have been recent studies suggesting a role for chronic inflammation in glioma, these studies are very limited and have not yielded uniform conclusions. We focused on the relationship between inflammatory markers in circulating blood and glioma diagnosis, grading and molecular typing, and assessed the relevance and validity of these markers in diagnosis, grading and molecular typing.

NLR is the most widely studied marker of inflammation in tumors, and it has been reported to associated with worse OS (41). Zheng et al. previously reported that preoperative NLR and dNLR levels in circulating blood were significantly higher in glioma patients than in healthy controls, the acoustic neuroma and meningioma groups (3). It has also been reported that dNLR has the highest sensitivity in differentiating gliomas from other intracranial tumors, and that NLR can be used as both a diagnostic parameter for GBM and a parameter for glioma grading (29). Our data showed similar results, indicating that NLR and dNLR were elevated in gliomas, with statistically significant differences compared to healthy controls, trigeminal neuralgia/hemifacial spasm, schwannoma, pituitary adenomas, and meningioma groups, and that NLR showed the highest efficacy in GBM versus healthy controls, with an AUC value of 0.9585 (0.9467- 0.9703). Additionally, our study demonstrated that NLR and dNLR were positively correlated with glioma grading, and NLR showed the highest sensitivity as a predictor of glioma grading with an AUC value of 0.8424 (0.8189-0.8660). Similar to other solid tumors, we suggest that enhanced neutrophil-mediated inflammatory response and reduced lymphocyte-mediated antitumor response in gliomas contribute to this result, with the degree of difference positively correlating with glioma grading. Mechanistically, it has been proposed that neutrophil infiltration in gliomas is caused by reactive oxygen species produced by glioma cells, neutrophil chemokines secreted by glioma cells, or ectopic expression of glioma stem cell markers (42–44). GBM cells have been reported to secrete a variety of immunosuppressive cytokines, such as IL-2, IL-4Rα, IL-10 and TGF-β to reduce lymphocyte infiltration (45–47). Meanwhile, it has also been reported that GBM can cause the loss of S1PR1 on the surface of T cells, thus confining a large number of T cells to the bone marrow and preventing them from exerting anti-tumor effects (48).

Despite the fact that PLR has been found to be a diagnostic and prognostic factor in a range of malignancies (20, 49, 50), reports on glioma have not yielded consistent results. Yang et al, reported that there is no significant correlation between PLR or absolute lymphocyte count and OS in patients with GBM (41). Han et al. (30). reported that elevated PLR levels were closely associated with poorer survival. Zheng et al. (3). reported no significant difference in PLR levels between patients with glioma and those with acoustic neuroma or meningioma, but in the glioma group, PLR levels were positively correlated with glioma grade. In our study, PLR levels were significantly higher in glioma patients than in controls and were positively correlated with glioma grade. PLR did not achieve satisfactory efficiency in the diagnosis of GBM compared with the healthy group, but its AUC value reached 0.6084 (0.5768-0.6399) for grade identification of GBM. However, there was no significant difference in platelet counts between glioma and other groups. We suggest that the increase in PLR is mainly due to the diminished lymphocyte-mediated anti-tumor response in glioma.

Several studies have found that low levels of LMR and PNI are poor prognostic indicators for a variety of malignancies (23, 51, 52). LMR and PNI levels in glioma patients were significantly lower than in healthy controls and other disease controls, and both were negatively correlated with glioma grade. We suggest that the infiltration of macrophages and the decrease of lymphocytes in glioma patients contributed to the decrease of LMR and that the decrease of the nutritional index PNI was related to the high intake of nutrients by glioma cells to maintain their own rapid proliferation. There is a large number of macrophages infiltration in the glioma microenvironment, which is associated with a variety of factors secreted by tumor cells, including IL-4, IL-6, IL-10, TGF-β, microRNAs, macrophage colony-stimulating factor (M-CSF), and CCL2. Interestingly, LMR was also significantly reduced in WHO I glioma patients, gradually decreasing with increasing glioma grade, suggesting that LMR may contribute to early detection of gliomas, which is similar to the results reported by Zheng et al. (3).

In peripheral tumors, there are established circulating serum or plasma biomarkers for the diagnosis of the patients or for monitoring recurrence (53, 54); but in gliomas, there are no well-established relevant markers available for glioma diagnosis due to the presence of the blood-brain barrier. Besides assessing the validity of preoperative circulating blood NLR, dNLR, PLR, LMR and PNI in the diagnosis of glioma using ROC curve analysis, we also analyzed their paired combinations. In our study, circulating blood NLR+dNLR and LMR+PNI were shown to be the most effective combination of markers for the diagnosis of GBM. Our data also confirmed that NLR and dNLR, LMR and PNI were positively correlated in GBM, respectively. The dual combined approach may be more appropriate for early glioma screening, and we believe that noninvasive, low-cost routine blood tests and liver function tests are feasible in early stages. For patients with glioma considered in neuroimaging, positive NLR+dNLR and LMR+PNI combined test is helpful for preoperative diagnosis, grading and molecular typing. The nutritional index PNI did not predict IDH status in gliomas, which leads us to speculate that potentially changes in IDH status do not change tumor metabolism. Our study also discovered that NLR and dNLR were the best markers for predicting IDH status in gliomas.

Our study also has some limitations, including the following: 1) there were only a small number of samples of controls other than meningiomas, suggesting that a larger study may be needed to confirm the findings; and 2) these changes in inflammatory markers were not specific to gliomas and may also be present in peripheral solid tumors.

## Conclusions

In conclusion, NLR and dNLR are of great value in the diagnosis of glioma, with NLR showing the highest accuracy in glioma diagnosis, grading and molecular typing. And we provide the first evidence that NLR+dNLR and LMR+PNI are highly sensitive and specific for the diagnosis and differential diagnosis of glioma, especially for the diagnosis of GBM and the differential diagnosis of GBM and low-grade glioma. Our study provides the theoretical basis for the development of non-invasive, low-cost tools for routine glioma screening.

## Data availability statement

The data analyzed in this study is subject to the following licenses/restrictions: the data was collected from our hospital.

Requests to access these datasets should be directed to FC, professorchen@foxmail.com.

## Ethics statement

The studies involving human participants were reviewed and approved by The Ethics Committee of Tangdu Hospital (TDLL-202210-04). Written informed consent to participate in this study was provided by the participants’ legal guardian/next of kin.

## Author contributions

Conceptualization: FC, MC, PJ, and YW; data curation and formal analysis: TH, PJ, and XX; funding acquisition: FC and LW; investigation: FC and XX; methodology: FC, PJ, YW, MC, PJ, and XX; project administration: LW; resources: FC, PJ, SG, YZ, YW, and NW; supervision: FC, XX, and LW; validation: FC and PJ; visualization: writing—original draft: FC; writing—review and editing: all authors contributed. All authors have reviewed and approved the manuscript.

## Funding

This research was funded by the Fund of Tang Du hospital (FC, No. 2021YFJH005 and No. 2021SHRC033).

## Conflict of interest

The authors declare that the research was conducted in the absence of any commercial or financial relationships that could be construed as a potential conflict of interest.

## Publisher’s note

All claims expressed in this article are solely those of the authors and do not necessarily represent those of their affiliated organizations, or those of the publisher, the editors and the reviewers. Any product that may be evaluated in this article, or claim that may be made by its manufacturer, is not guaranteed or endorsed by the publisher.

## Supplementary material

Supplementary Figure 1 Correlation of preoperative inflammatory markers. A. LMR vs NLR, LMR vs dNLR, PNI vs PLR, PNI vs NLR, PNI vs dNLR, PNI vs LMR in Glioma; B. LMR vs dNLR, PNI vs dNLR, LMR vs NLR, LMR vs PLR, PNI vs PLR, PNI vs NLR in GBM.

Supplementary Figure 2 The diagnostic value of preoperative inflammatory markers in glioma diagnosis and glioma grading.

Supplementary Figure 3 Influences on preoperative inflammatory markers PNI caused by IDH1 mutation within glioma grade.
