# Supplementary figures and images for: The role of preoperative inflammatory markers in patients with central nervous system tumors, focus on glioma
Source: Front Oncol. 2022 Nov 22;12:1055783. doi: 10.3389/fonc.2022.1055783 (PMC9723353; doi:10.3389/fonc.2022.1055783)

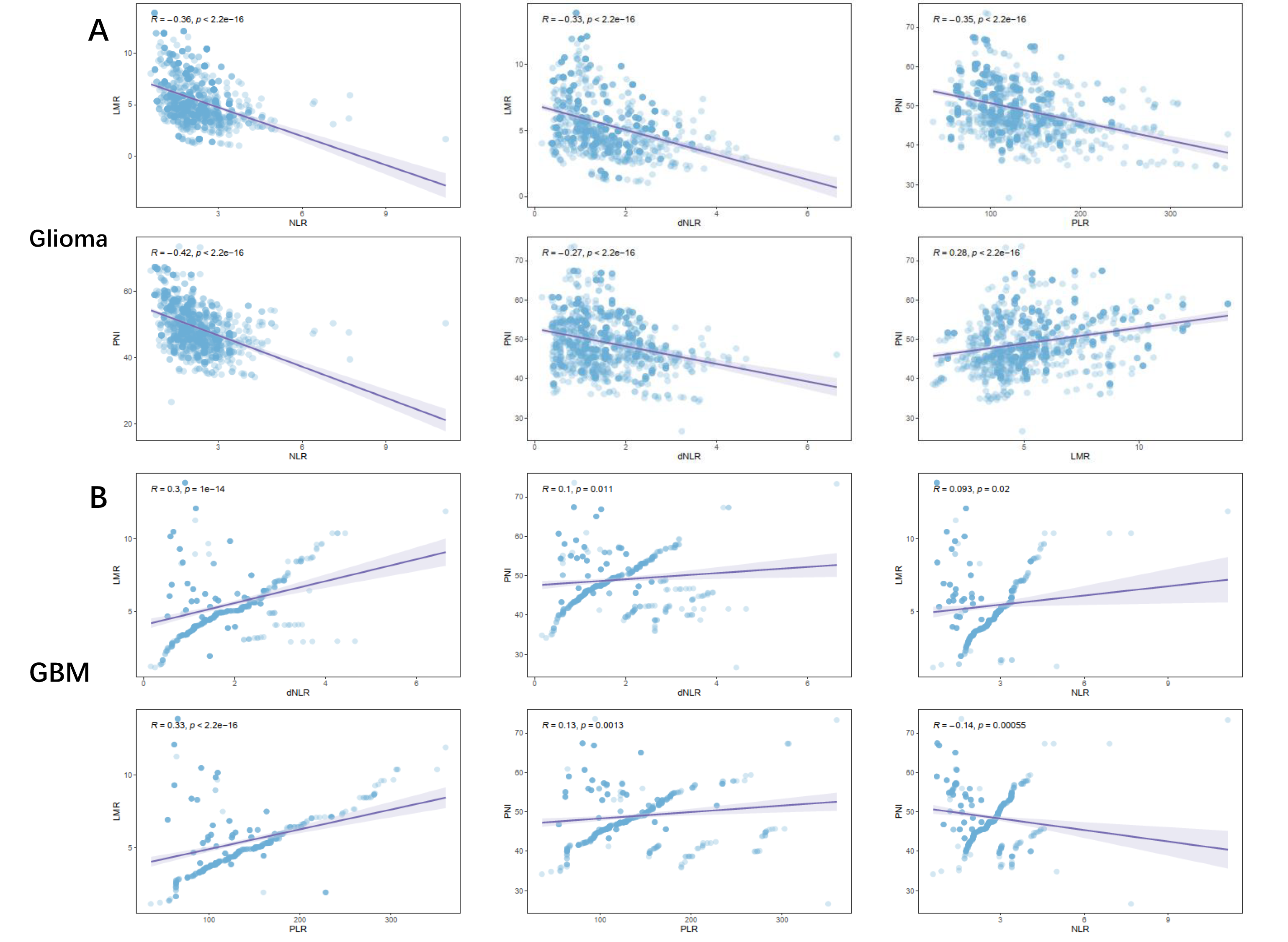

Supplement: Supplementary Figure 1 — Correlation of preoperative inflammatory markers. A. LMR vs NLR, LMR vs dNLR, PNI vs PLR, PNI vs NLR, PNI vs dNLR, PNI vs LMR in Glioma; B. LMR vs dNLR, PNI vs dNLR, LMR vs NLR, LMR vs PLR, PNI vs PLR, PNI vs NLR in GBM. [file Image_1.tif]

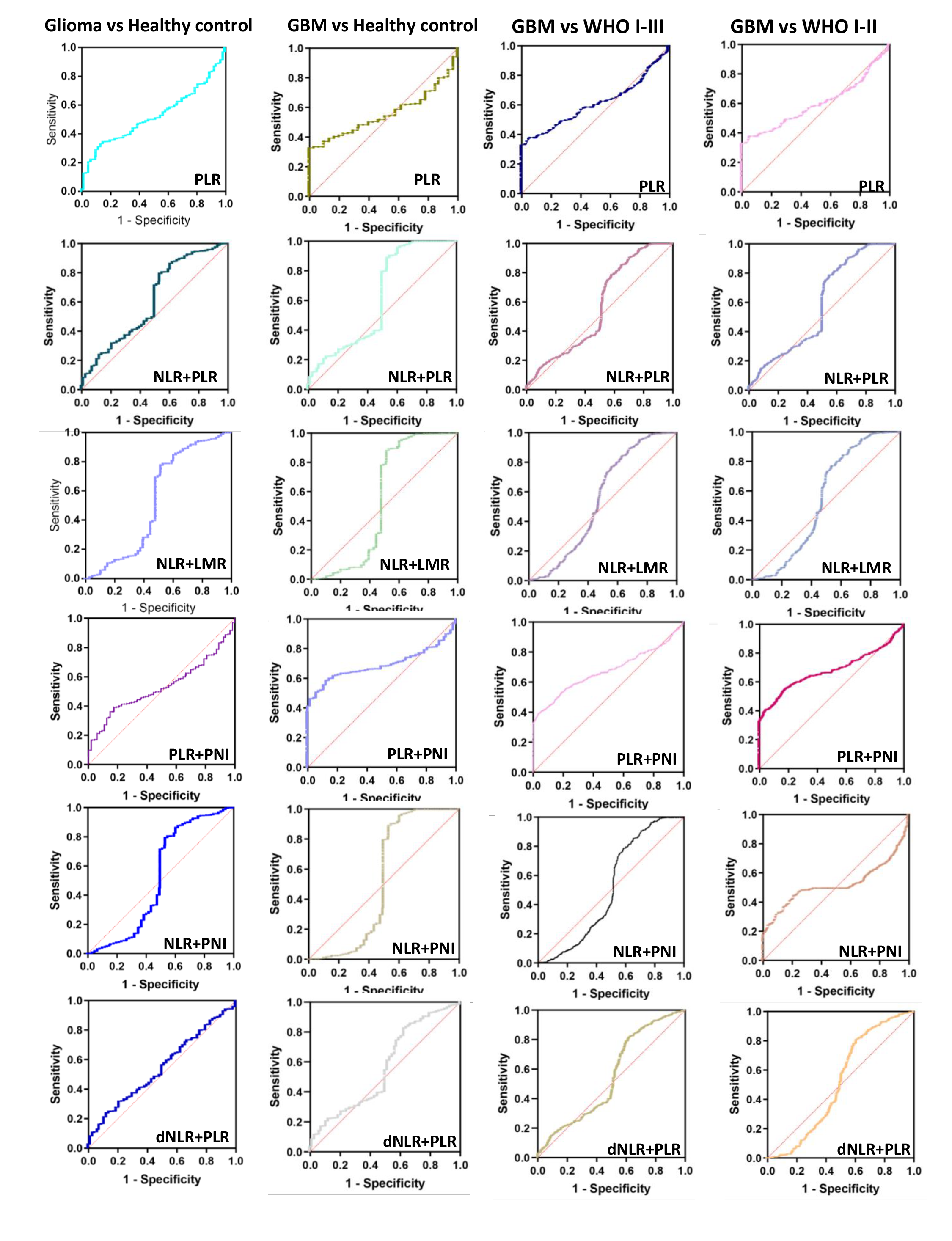

Supplement: Supplementary Figure 2 — The diagnostic value of preoperative inflammatory markers in glioma diagnosis and glioma grading. [file Image_2.tif]

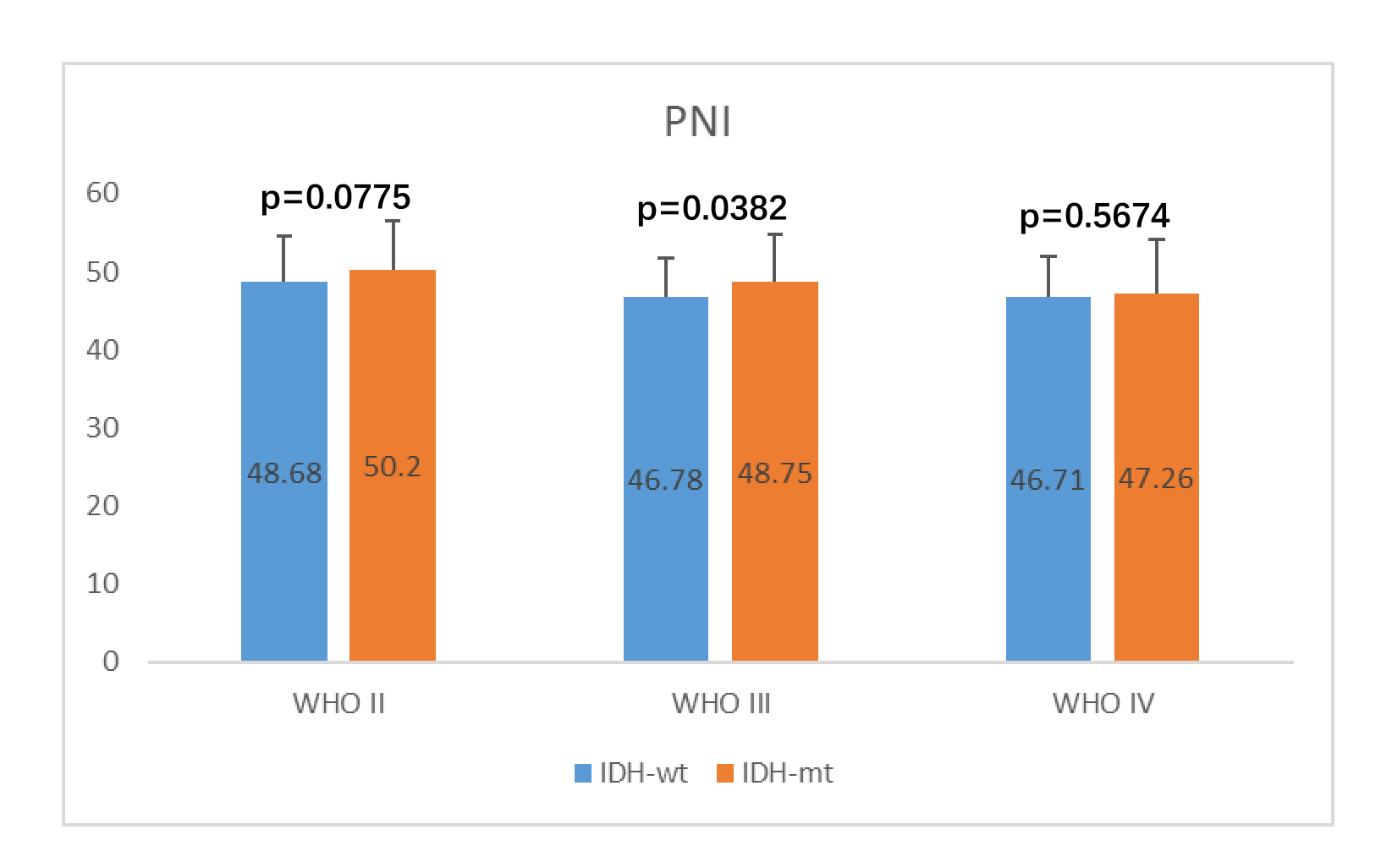

Supplement: Supplementary Figure 3 — Influences on preoperative inflammatory markers PNI caused by IDH1 mutation within glioma grade. [file Image_3.tif]
